# Unculturable and culturable periodontal-related bacteria are associated with periodontal inflammation during pregnancy and with preterm low birth weight delivery

**DOI:** 10.1038/s41598-020-72807-9

**Published:** 2020-09-25

**Authors:** Changchang Ye, Zhongyi Xia, Jing Tang, Thatawee Khemwong, Yvonne Kapila, Ryutaro Kuraji, Ping Huang, Yafei Wu, Hiroaki Kobayashi

**Affiliations:** 1 State Key Laboratory of Oral Diseases & National Clinical Research Center for Oral Diseases & Dept. of Periodontology West China Hospital of Stomatology, Sichuan University, Chengdu, China; 2 Department of Periodontology, Graduate School of Medical and Dental Sciences, Tokyo Medical and Dental University (TMDU), 1-5-45 Yushima, Bunkyo-ku, Tokyo, 113-8549 Japan; 3 Division of Periodontology, Department of Orofacial Sciences, School of Dentistry, University of California San Francisco, San Francisco, CA USA; 4 Department of Life Science Dentistry and Department of Periodontology, The Nippon Dental University School of Life Dentistry at Tokyo, Tokyo, Japan; 5 Sumitomo Corporation Dental Clinic, Tokyo, Japan

**Keywords:** Risk factors, Gingivitis, Periodontitis

## Abstract

Recent studies revealed culturable periodontal keystone pathogens are associated with preterm low birth weight (PLBW). However, the oral microbiome is also comprised of hundreds of ‘culture-difficult’ or ‘not-yet-culturable’ bacterial species. To explore the potential role of unculturable and culturable periodontitis-related bacteria in preterm low birth weight (PLBW) delivery, we recruited 90 pregnant women in this prospective study. Periodontal parameters, including pocket probing depth, bleeding on probing, and clinical attachment level were recorded during the second trimester and following interviews on oral hygiene and lifestyle habits. Saliva and serum samples were also collected. After delivery, birth results were recorded. Real-time PCR analyses were performed to quantify the levels of periodontitis-related unculturable bacteria (*Eubacterium saphenum*, *Fretibacterium* sp. human oral taxon(HOT) 360, *TM7* sp. HOT 356, and *Rothia dentocariosa), and cultivable bacteria (Aggregatibacter actinomycetemcomitans, Porphyromonas gingivalis, Tannerella forsythia*, *Treponema denticola*, *Fusobacterium nucleatum* and *Prevotella intermedia)* in saliva samples. In addition, ELISA analyses were used to determine the IgG titres against periodontal pathogens in serum samples. Subjects were categorized into a Healthy group (H, n = 20) and periodontitis/gingivitis group (PG, n = 70) according to their periodontal status. The brushing duration was significantly lower in the PG group compared to the H group. Twenty-two of 90 subjects delivered PLBW infants. There was no significant difference in periodontal parameters and serum IgG levels for periodontal pathogens between PLBW and healthy delivery (HD) groups. However, ordinal logistic regression analysis revealed that a higher abundance of *Treponema denticola, Prevotella intermedia, Fretibacterium* sp. HOT360 and lower levels of *Rothia dentocariosa* were significantly associated with the presence of periodontal disease during pregnancy. Moreover, the amount of *Eubacterium saphenum* in saliva and serum IgG against *Aggregatibacter actinomycetemcomitans* were negatively correlated with PLBW. Taken together, unculturable periodontitis-associated bacteria may play an important role both in the presence of periodontal inflammation during pregnancy and subsequent PLBW.

## Introduction

The oral microbiome plays an essential role both in the development of normal oral physiology and host defense^1^. Dysbiosis of the oral microbiome leads to oral diseases and is also associated with the progression of systemic diseases, such as cardiovascular disease^2^, diabetes mellitus^3^, and preterm low birth weight (PLBW)^4^.

Recent molecular techniques have revealed that several culturable bacteria of the oral microbiome, such as *Aggregatibacter actinomycetemcomitans (A. actinomycetemcomitans), Porphyromonas gingivalis (P. gingivalis), Tannerella forsythia (T. forsythia)*, *Treponema denticola (T. denticola)*, *Fusobacterium nucleatum (F. nucleatum)* and *Prevotella intermedia(P. intermedia)* are recognized as key periodontal pathogens^5,6^ and they have been used as diagnostic markers of periodontitis^7,8^. However, the oral microbiome is also comprised of hundreds of ‘difficult-to culture’ or ‘not-yet-cultivable’ bacterial species, and their functions remain unknown^9^.

A growing body of literature has suggested that there is a link between periodontal disease and preterm low birth weight (PLBW). Most of these studies focused on the roles of culturable periodontal pathogens, and no study has specifically investigated the role of unculturable bacterial species in PLBW. Four unculturable or difficult-to-culture bacterial species *Eubacterium saphenum (E. saphenum),* *Rothia dentocariosa (R. dentocariosa)*, *Fretibacterium* sp. HOT 360 and *TM7* sp. HOT 356 have been commonly identified in the oral microbiome of periodontitis patients and these bacteria have been identified as opportunistic pathogens and are associated with periodontal disease and infections^10–13^.

Given the potential importance of unculturable periodontal-associated bacteria and limited knowledge about their role in periodontal disease and PLBW outcomes, we investigated this topic in the current study. We determined the levels of four unculturable or difficult-to-culture periodontitis-associated bacterial species and six culturable periodontal pathogens in saliva samples collected from Chinese pregnant women with or without periodontal disease. In addition, the association of PLBW with maternal periodontal conditions and serum IgG titers against periodontal pathogens were evaluated.

## Results

### Subject characteristics in the healthy and periodontitis/gingivitis groups

The duration of tooth brushing in the Periodontitis/Gingivitis group was significantly shorter than that in the Healthy group (Table 1). There were no significant differences between the two groups in terms of socio-economic characteristics or serum levels of high-sensitivity C-reactive protein (hs-CRP).

**Table 1:** Subject characteristics in healthy and periodontitis/gingivitis groups.

	H (n = 20)	PG (n = 70)	*P*-value
Age	28.8 ± 2.9	29.1 ± 4.4	NS
Primiparity (%)	90.0 (18/20)	77.1 (54/70)	NS
Alcohol consumption history (%)	5.0 (1/20)	4.3 (3/70)	NS
Smoking history (%)	0	0	NS
**Oral hygiene behavior**			
Tooth brushing frequency/day	2.2 ± 0.5	2.1 ± 0.4	NS
Duration of tooth brushing (min)	2.7 ± 1.3	2.3 ± 0.9*	0.03
Dental floss or interproximal brush utilization (yes %)	25.0 (5/20)	20.0 (14/70)	NS
Mouth rinse utilization (yes %)	35.0 (7/20)	32.9 (23/70)	NS
**Education and socio-economic characteristics**			
Education ≥ 12 years (yes %)	90.0 (18/20)	78.6 (55/70)	NS
Income ≥ local average salary (yes %)	80.0 (16/20)	62.9 (44/70)	NS
Health insurance (yes %)	60.0 (12/20)	62.9 (44/70)	NS
**Periodontal clinical parameters**			
Mean PPD (mm)	1.8 (1.7 2.0)	2.6 (2.3 2.9)*	< 0.0001
Mean CAL (mm)	1.8 (1.7 2.1)	2.6 (2.3 3.0)*	< 0.0001
PPD ≥ 3 mm %	13.1 (9.8 20.1)	52.1 (36.3 64.3)*	< 0.0001
PPD ≥ 4 mm %	0.6 (0 2.23)	11.9 (6.5 21.9)*	< 0.0001
PPD ≥ 5 mm %	0	2.1 (0.6 5.7)*	< 0.0001
BOP %	12.5 (7.5 19.4)	53.6 (41.1 73.7)*	< 0.0001
**Birth outcomes**			
Birth weeks	39.0 ± 1.5	39.0 ± 1.5	NS
Birth weight (g)	3241.2 ± 456.6	3155.9 ± 457.6	NS
Caesarean section (%)	65.0 (13/20)	48.6 (34/70)	NS
PB (%)	5.0 (1/20)	8.6 (6/70)	NS
SGA (%)	10.0 (2/20)	18.6 (13/70)	NS
**Systemic inflammatory mediator**			
hs-CRP (μg/mL)	3.5 (1.1 9.9)	5.0 (2.7 7.2)	NS

Parametric continuous variables were tested using an unpaired t-test and are given as means ± standard deviations; nonparametric continuous variables were tested using the Mann–Whitney U-test and are given as medians (quartile).

PG, periodontitis/gingivitis; PB, preterm birth; SGA, small for gestational age; PPD, pocket probing depth; CAL, clinical attachment loss; BOP, bleeding on probing; hs-CRP, high-sensitivity C-reactive protein; NS, not significant.

*Significantly different from the H group (*P* < 0.05).

**Figure 1:**
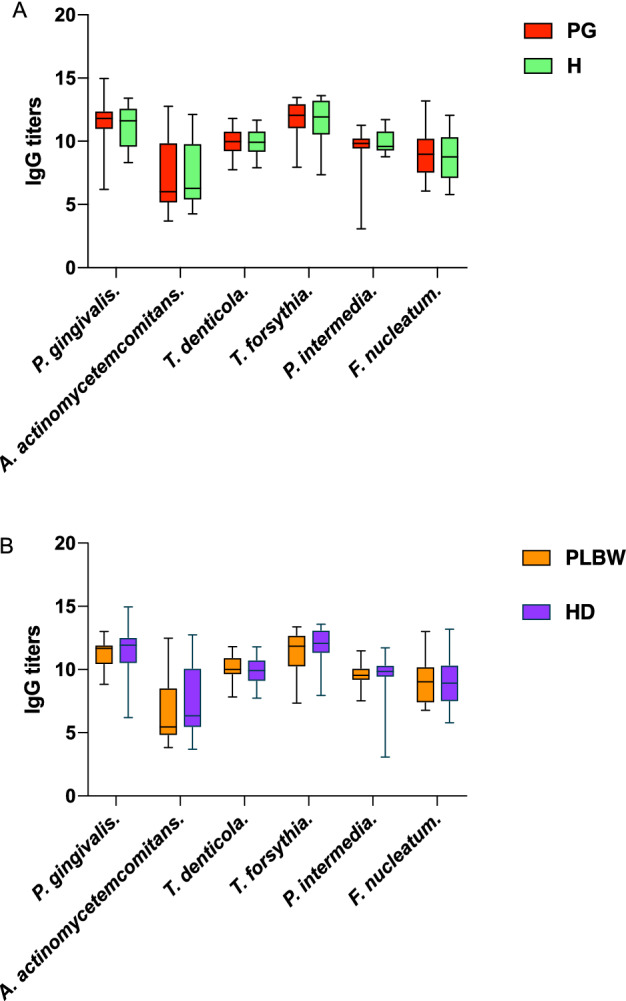
The comparison of IgG titers against periodontal pathogens in groups. The box plots represent the levels of IgG titers against periodontal pathogens evaluated in this study. (**A**) the comparison of IgG titers between healthy and Periodontitis/Gingivitis groups; (**B**) the comparison of IgG titers between HD and PLBW groups. The Mann–Whitney U-tests were applied to test the differences between groups; *P < 0.05.

There were no significant differences between the two groups in IgG antibodies against periodontal pathogens (Fig. 1A and Table S1).

### Comparison of bacterial load between healthy and periodontitis/gingivitis groups

**Figure 2:**
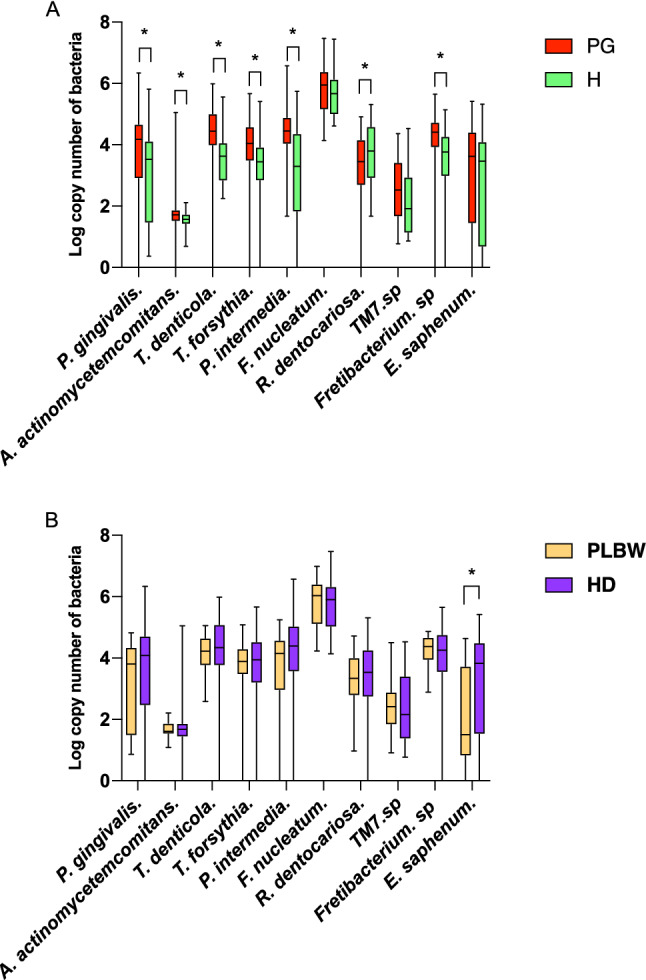
The comparison of bacteria load in groups. The box plots represent the levels of periodontitis-associated bacterial species evaluated in this study. (**A**) The comparison of bacteria load between healthy and Periodontitis/Gingivitis groups. (**B**) The comparison of bacteria load between HD and PLBW groups. The Mann–Whitney U-tests were applied to test the differences between groups; *P < 0.05.

As showed in Fig. 2A and Table S2, the amount of *P. gingivalis, A. actinomycetemcomitans, T. forsythia, T. denticola*, *P. intermedia* and *Fretibacterium* sp. HOT 360 in saliva in the PG group were significantly higher than those in the H group. In contrast, *R. dentocariosa* was significantly lower in the PG group.

### Correlation of periodontal parameters with bacterial copy number

**Figure 3:**
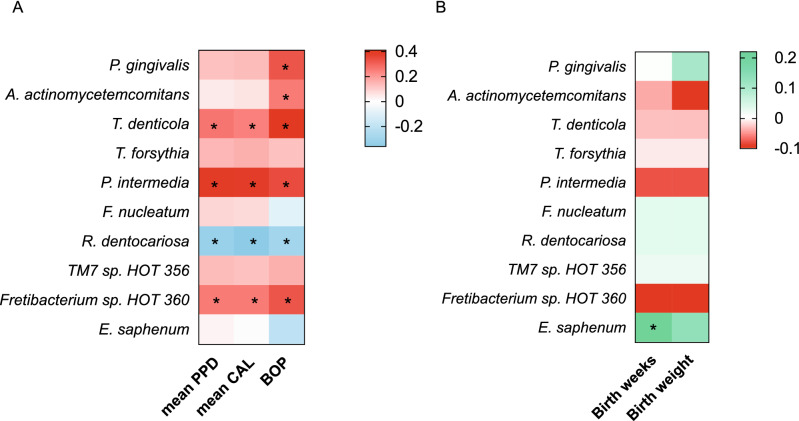
Correlation of bacteria load with periodontal parameters and birth results. (**A**) The heat map of Pearson's correlation coefficient between periodontitis-associated bacteria and periodontal parameters. negative: blue; positive: red; (**B**) the heat map of Pearson's correlation coefficient between periodontitis-associated bacteria and birth results. negative: red; positive: green. Correlation between bacterial load and periodontal parameters, birth results were analyzed by a Pearson's correlation coefficient test; *P < 0.05.

Figure 3A and Table S3 shows the results of the correlation analysis between periodontal parameters and bacterial copy number (log scale). The number of *P. gingivalis* and *A. actinomycetemcomitans* were positively correlated with BOP positive sites (%). Moreover, the bacterial load of *P. intermedia, T. denticola* and *Fretibacterium* sp. HOT 360 in saliva were positively correlated with mean PPD, mean CAL, and BOP positive sites (%). On the other hand, *R. dentocariosa* was negatively correlated with mean PPD, mean CAL, and BOP positive sites (%).

### Subject characteristics in healthy delivery and preterm low birth weight groups

Seven of 90 pregnant women delivered PB infants and the other fifteen individuals delivered SGA infants. There was no significant difference in the gestational week at birth (birth week), birth weight, incidence (%) of PB, and SGA between the Healthy and Periodontitis/Gingivitis groups (Table 1).

Table 2 shows the subject characteristics of the HD and PLBW groups. There were no significant differences in oral hygiene behaviors, socio-economic characteristics, and serum cytokine levels against periodontal pathogens between the two groups. There were no significant differences between the two groups in IgG antibodies against periodontal pathogens (Fig. 1B and Table S4).

**Table 2:** Subjects’ characteristic in health delivery group and preterm low birth weight group.

	HD (n = 68)	PLBW (n = 22)	*P*-value
Age	28.8 ± 3.9	29.7 ± 4.8	NS
Primiparity (%)	78.0 (53/68)	90.9 (20/22)	NS
Alcohol consumption history (%)	4.4 (3/68)	4.5 (1/22)	NS
Smoking history (%)	0	0	NS
**Oral hygiene behavior**			
Tooth brushing frequency/day	2.1 ± 0.5	2.2 ± 0.4	NS
Duration of tooth brushing (min)	2.4 ± 1.1	2.3 ± 0.9	NS
Dental floss or interproximal brush utilization (yes %)	23.5 (16/68)	13.6 (3/22)	NS
Mouth rinse utilization (yes %)	35.3 (24/68)	27.3 (6/22)	NS
**Education and social economic characteristic**			
Education ≥ 12 years (yes %)	82.4 (56/68)	77.3 (17/22)	NS
Income ≥ local average salary (yes %)	67.6 (46/68)	63.6 (14/22)	NS
Health insurance (yes %)	60.3 (41/68)	68.2 (15/22)	NS
**Periodontal clinical parameters**			
Mean PPD (mm)	2.4 (2.1 2.8)	2.5 (2.2 2.8)	NS
Mean CAL (mm)	2.5 (2.1 2.9)	2.6 (2.2 3.0)	NS
PPD ≥ 3 mm %	41.5 (23.2 61.8)	52.0 (29.2 63.3)	NS
PPD ≥ 4 mm %	9.3 (4.2 17.9)	11.0 (4.5 19.8)	NS
PPD ≥ 5 mm %	1.2 (0.2 5.1)	0.6 (0 5.7)	NS
BOP %	44.6 (29.0 69.2)	46.4 (40.3 80.7)	NS
**Birth outcomes**			
Birth week	39.4 ± 1.0	37.9 ± 2.0*	< 0.001
Birth weight (g)	3360.3 ± 351.5	2601.8 ± 181.2*	0.004
PB (%)	0	31.8 (7/22)*	< 0.001
SGA (%)	0	68.2 (15/22)*	< 0.001
Caesarean section (%)	51.5 (35/68)	54.5 (12/22)	NS
**Systemic inflammation mediator**			
CRP (μg/mL)	3.9 (1.8 7.1)	5.5 (3.0 7.8)	NS

Parametric continuous variables were tested using an unpaired t-test and are given as means ± standard deviations; nonparametric continuous variables were tested using the Mann–Whitney U-test and are given as medians (quartile).

PB, preterm birth; SGA, small for gestational age; PPD, pocket probing depth; CAL, clinical attachment loss; BOP, bleeding on probing; hs-CRP, high-sensitivity C-reactive protein; NS, not significant..

*Significantly different from the HD group (*P* < 0.05).

### Comparison of bacterial load between healthy delivery and preterm low birth weight groups

Aa showed in Fig. 2B and Table S5, the amount of *E. saphenum* was significantly lower in the PLBW group than that in the HD group (p < 0.01). There was no other significant difference in periodontal pathogens between the two groups.

### Correlation of birth results with log copy number of bacteria

As showed in Fig. 3B and Table S6, the log copy number of *E. saphenum* was positively correlated with the gestational week at birth (r = 0.22, *p* = 0.03). There was no other significant difference between bacteria and birth results.

### Potential risk indicators for periodontal disease during pregnancy and PLBW

Ordinal logistic regression analysis revealed that the high levels of *T. denticola, P. intermedia, Fretibacterium* sp. HOT360 and low amount of *R. dentocariosa* in saliva were significantly associated with the presence of gingival inflammation during pregnancy (Table 3). Meanwhile, the amount of *E. saphenum* and serum IgG against *A. actinomycetemcomitans* showed a negative correlation with the prevalence of PLBW (Table 4, p < 0.05).

**Table 3:** Potential risk indicators for periodontal disease during pregnancy in all subjects.

	Odds ratio	95% confidence interval	*P* value
*T. denticola*	3.6*	1.3–9.5	0.003
*P. intermedia*	1.9*	1.02–3.5	0.043
*Fretibacterium* sp. HOT 360	2.3*	1.04–5.3	0.040
*R. dentocariosa*	0.3*	0.1–0.7	0.009

* Statistically significant p < 0.05; ordinal logistic regression analysis.

**Table 4:** Potential risk indicators for PLBW in all subjects.

	Odds ration	95% confidence interval	*P* value
*E. saphenum*	0.6*	0.39–0.98	0.01
Anti-*A. actinomycetemcomitans* IgG	0.7*	0.56–0.98	0.03

*Statistically significant p < 0.05; ordinal logistic regression analysis.

## Discussion

The oral microbiome can be affected by numerous environmental factors including pH, anaerobic conditions, nutrition, and hormone levels^14–16^. During pregnancy, periodontal tissues show an enhanced inflammatory response to plaque microbiome, believed to be mediated by female sex hormones^17^ . Periodontal pathogens, such as *P. intermedia, P. gingivalis, T. forsythia, Campylobacter rectus, F. nucleatum*, and *A. actinomycetemcomitans* are highly abundant in the saliva and subgingival biofilm of pregnancy gingivitis patients^18–20^. In addition, *P. gingivalis*, *T. denticola*, *Fretibacterium* sp., and *P. intermedia* levels in saliva and plaque samples from pregnant women were associated with gingival bleeding^21^. Similarly, in the present study, *P. gingivalis, A. actinomycetemcomitans, P. intermedia, T. denticola* and *Fretibacterium* sp. HOT 360 levels were positively correlated with periodontal parameters during pregnancy (Fig. 3A and Table S3). In the current study, although IgG antibodies against periodontal pathogens were present at higher levels in the PG group, there was no significant difference between the H and PG groups.

*Fretibacterium* sp. HOT 360, which belongs to phylum Synergistetes, is found in high proportions in subgingival plaque or saliva samples of patients with chronic periodontitis^13,22–25^. However, the characteristics of this bacterial species remain unknown. The present study findings and that of our previous report^13^, suggest that *Fretibacterium* sp. HOT 360 may be a periodontal disease-related bacteria, and it was positively correlated with a deterioration of maternal gingival status.

*R. dentocariosa* is a gram-positive bacterium that is representative of the normal flora in the human oral cavity and pharynx^22,26^. Kumar et.al.^27^ also reported that *R. dentocariosa* exists in high proportions under healthy gingival conditions. In addition, our study demonstrated that there was a negative correlation between *R. dentocariosa* and periodontal parameters, such as PPD and BOP (Fig. 3A), and a decreased amount of *R. dentocariosa* was associated with an increased risk for gingivitis in pregnant women (Table 3). This suggests that a decrease in *R. dentocariosa* may be a potential risk indicator for and play a role in the onset of gingivitis in pregnancy. However, *R. dentocariosa* has also been isolated from dental caries^28–30^. Taken in aggregate, these findings suggest that *R. dentocariosa* may play a role in the development of dental caries, but decreasing levels of *R. dentocariosa* may predispose pregnant women to the development of gingivitis.

*Eubacterium saphenum* is a newly discovered anaerobic gram-positive and rod-shaped bacteria, which was reported as a putative periodontal pathogen, as it was isolated from periodontal pockets in periodontitis patients^10,22,31^. No study has addressed the role of *E. saphenum* in systemic diseases. In this study, the amount of *E. saphenum* in saliva did not show any significant differences between the H and PG groups. However, a decreased amount of *E. saphenum* was correlated with PLBW (Table 4). Furthermore, the amount of *E. saphenum* was positively correlated with the birth week versus the birth weight (Fig. 3B and Table S6). This indicated the lacking of *E. saphenum* might be related to the progression of preterm birth than intrauterine growth restriction. A possible mechanism for this finding may be that there is a loss of a beneficial effect in controlling an oral microbiome dysbiosis caused by a reduction in *E. saphenum* that subsequently induces an altered maternal and fetal immunological response and systemic inflammation, leading to preterm birth. Further investigation is warranted to reveal the potential mechanism. Also, the lack of IgG against *A. actinomycetemcomitans* was associated with an increased risk for PLBW. Maternal IgG might protect the fetus from exposure to pathogens^32,33^. Taken together, these data suggest that loss of *E. saphenum* and loss of IgG response to *A. actinomycetemcomitans* may be a risk indicator for PLBW.

Although brushing frequency was similar in the Healthy and Periodontitis/Gingivitis group, the shorter brushing time for the PG group might contribute to the development of periodontal inflammation during pregnancy in this group. Hence, proper personal plaque control measures and periodontal therapy should be pursued to reduce periodontal inflammation and increases in periodontitis-associated bacteria during pregnancy.

The present investigation is the first study to explore the relationship between unculturable periodontitis-associated bacteria and PLBW. However, this study has some limitations, including an uneven distribution of subjects in the H and PG groups. A second limitation is its somewhat cross-sectional design. The microbial etiology of maternal periodontitis appears to be complex, and this fact warrants further large-scale longitudinal studies to understand these bacteria and their role in PLBW outcomes.

In conclusion, the results of our study showed that a greater abundance of *T. denticola, P. intermedia, Fretibacterium* sp. HOT360 and a lower abundance of *R. dentocariosa* in saliva may pose an increased risk for gingival/periodontal inflammation during pregnancy. Moreover, a low amount of *E. saphenum* in saliva and anti-*A. actinomycetemcomitans* IgG in serum may increase the risk of PLBW.

## Methods

### Ethics statement

This study and related consent procedures were approved by the Institution Ethics Committee of West China Hospital of Stomatology, Sichuan University (No WCHSIRB-OT-2016-053) in alignment with the 1964 Helsinki declaration and its later amendments on comparable ethical standards. Written informed consent was obtained from all human subjects who participated in this investigation.

### Study population

The present study was a longitudinal observational study. The optimal sample size was determined using the G*Power software^34^. The tail was set at one, the odds ratio at 2, Pr(Y = 1/X = 1)HO at 0.2, alpha error at 0.05, and power (1 – beta) at 0.8, R^2^ other X at 0, X distribution at normal, X parm μ at 0 and X parm σ at 1 for logistic regression, and the minimum sample size was estimated to be 88 participants.

Ninety Chinese pregnant women aged 21 to 39 who received a periodontal examination during their middle trimester of pregnancy were recruited in the Department of Periodontology, West China Dental Hospital, Sichuan University from May 2015 to May 2018. All subjects had a minimum of 20 teeth and did not receive any periodontal treatment or antibiotic treatment within three months of their participation in the study. Any subjects with systemic disease or multiple gestations were excluded. All patients were divided into a periodontitis/gingivitis group (PG; n = 70) and gingival health group (H; n = 20) according to their periodontal status (described subsequently). After delivery, gestational age at birth, birth weight, infant gender, and delivery mode were recorded. Preterm birth (PB) was defined as a gestational age less than 37 weeks and small for gestational age (SGA) delivery was defined as a birth weight of less than the 10th percentile for gestational age based on Chinese norms^35^. Therefore, the PLBW group consisted of PB and/or SGA. The healthy delivery (HD) group was defined as pregnancy’s showing a gestational age ≥ 37 weeks and a birth weight of more than the 10th percentile for gestational age.

### Interview and periodontal examination

All pregnant women underwent a full-mouth periodontal examination at 26 to 28 weeks of gestation. Before the examination, information about confounding factors, such as oral hygiene behavior (the frequency of tooth brushing, duration of tooth brushing, utilization of special appliances, and utilization of an oral rinse), smoking habits, socioeconomic characteristics (education level, income level, and health insurance status), and an alcohol consumption habits was obtained by structured interviews.

Periodontal examinations were performed by one periodontal specialist (C.Y.) with the use of a manual probe (PCP-UNC15, Hu-Friedy, Chicago, USA). The periodontal parameters, including pocket probing depth (PPD), clinical attachment level (CAL), and bleeding on probing (BOP), were recorded at all six sites for each tooth (mesiobuccal, mid-buccal, distobuccal, mesiolingual, mid-lingual and distolingual). Subsequently, subjects were divided into a periodontitis/gingivitis (PG) group (n = 70) and a Healthy (H) group (n = 20) according to periodontal status. Subjects showing BOP in > 25% of sites and/or ≥ 4 teeth with one or more sites of PPD ≥ 4 mm were placed in the PG group. Subjects who did not fit the above criteria were diagnosed as having clinical gingival health and were placed in the H group^36–38^.

### Sample collection

One mL of unstimulated saliva was obtained at the same time as the periodontal examination and then stored at − 80 °C. Participants were instructed to abstain from food intake and oral hygiene procedures for at least 2 h before saliva collection. Also, at 28 weeks of gestational age, peripheral blood samples were collected and centrifuged at 1500×*g* for 10 min at 4 °C. Subsequently, serum samples were collected and immediately stored at − 80 °C until further analysis.

### Bacterial detection in saliva

Genomic DNA was extracted using the QIAamp DNA Mini kit (QIAGEN., CA, USA). The amount of specific bacterial species in duplicate saliva samples were then quantified by real-time polymerase chain reaction (RT-PCR) using primers and TaqMan probes for *E. saphenum*, *R. dentocariosa,* *Fretibacterium* sp. HOT 360, *TM7* sp. HOT 356, *A. actinomycetemcomitans*, *P. gingivalis*, *T. forsythia, T. denticola, F. nucleatum,* and *P. intermedia*^13,39–41^ (Table 5). The PCR reaction was performed in a total volume of 20 μL of reaction mixtures containing 10 μL Premix Ex Taq, 0.4 μL each of the forward and reverse primer (final concentration 10 nM), 0.5 μL TaqMan probe (final concentration 10 nM), 1 μL template DNA solution, and an appropriate volume of sterilized DNase- and RNase- free water. The PCR TaqMan Master Mix without DNA was used as negative control.

**Table 5:** PCR primers and probes for periodontal pathogens and “not-yet-culturable” periodontitis-related bacteria.

Bacteria	Primer	Probe
*P. gingivalis*^39^	F:TAGCTTGCTAAGGTCGATGG	TGCGTAACGCGTATGCAACTTGCC
	R:CAAGTGTATGCGGTTTTAGT	
*A. actinomycetemcomitans*	F:TCTTACCTACTCTTGACATCCGAA	AGAACTCAGAGATGGGTTTGTGCCTTAG
	R:ATGCAGCACCTGTCTCAAAGC	
*T*. *forsythia*	F:CGACGGAGAGTGAGAGCTTTCT	CGTCTATGTAGGTGCTGCATGGTTGTCG
	R:GCGCTCGTTATGGCACTTAAG	
*P. intermedia*	F:CCGCCTAATACCCGATGTTG	CACATATGGCATCTGACGTGGACCAAA
	R:CCCATCCTCCACCGATGA	
*F. nucleatum*	F:GTCAGGATGAGAAATCTAAGGC	GAAGAGGAGCCCTTGTGTGTGAGTATAC
	R:CCTCGTGCGCTTTGTATC	
*T. denticola*	F:AGAGCAAGCTCTCCCTTACCGT	CAGCGTTCGTTCTGAGCCAGGATCA
	R:TAAGGGCGGCTTGAAATAATAATG	
*Fretibacterium* sp. HOT 360^13^	F:GGAAACATTGACGACGCTG	CACCTGTGTATGCTCACTGCCCGAAA
	R:CTTAACCCAACATCTCACGAC	
*TM7* sp. HOT 356^13^	F:TGACTGGGCGTAAAGAGTTG	TCGCTCGCTAACTTGACCGCC
	R:TTCGAACAACAAGCTATCGG	
*R. dentocariosa*^40^	F:CTGGGCAAAGCGTCTGGAAAAC	AGCAAGACCGGATTTCTCAGGTACAGAGGTGTCA
	R:GAAATCACGAATCGGGGAAATCTC	
*E. saphenum*^40^	F:GAAGGCCTTTGGGTTGTAAG	TGCCAGCAGCCGCGGTAATA-TAMRA
	R:CCCAATAATTCCGGATAACG	

F, forward; R, reverse.

The optimized thermal cycling conditions for amplification were carried out on a thermal cycler device (Real-Time System II; Takara-bio, Tokyo, Japan) at 95 ºC for 30 s, 95 ºC for 5 s (40 cycles) and 60 ºC for 30 s. Standard curves were obtained by using tenfold serial dilutions of artificial synthetic genes, which contained the amplified region corresponding to 16S sequences of each bacteria (Eurofin genomics, Tokyo, Japan).

### Detection of serum IgG antibody titres

Serum antibody titers of IgG against the culturable periodontal pathogens including *A. actinomycetemcomitans*, *P. gingivalis*, *T. forsythia, T. denticola, F. nucleatum and P. intermedia* were determined using an enzyme-linked immunosorbent assay (ELISA) according to a previously described method^42^. The sonication-extracted antigens of *P. gingivalis* (ATCC 33277), *A. actinomycetemcomitans* (ATCC 43719), *P. intermedia* (ATCC 25611) and *T. forsythia* (ATCC 43037) were prepared by the method of Yano-Higuchi et al.^43^. Similarly, *F. nucleatum* (ATCC 25586) and *T. denticola* (ATCC 35405) antigens were prepared by previously described methods^44,45^.

### Detection of high-sensitivity C-reactive protein (hs-CRP) in serum

Serum levels of hs-CRP were measured using a commercial ELISA kit according to the protocol supplied by the manufacturer (Helica CRP assay, Helica Biosystems, Inc.).

### Statistical analysis

Statistical analysis was performed using SPSS 20.0 software (SPSS Inc., Chicago, IL, USA). Initially, the distribution pattern of the data was assessed using the Shapiro–Wilk test. Thereafter, an unpaired t-test was used to analyze the statistical differences in age, brushing frequency, brushing time, weeks at birth, and birth weight between the H and PG groups, or HD and PLBW groups, respectively. In addition, a Mann–Whitney U-test was used to analyze the mean PPD, mean CAL, sites with PPD ≥ 3 mm (%), sites with PPD ≥ 4 mm (%), sites with PPD ≥ 5 mm (%), BOP-positive sites (%), serum CRP concentration, the number of bacteria in saliva, and the serum IgG titer between the H and PG groups, or HD and PLBW groups. Also, a chi-squared test was used to analyze the primiparity rate, the utilization rate of dental floss or interproximal brushing, the utilization rate of mouth rinse, the amount showing more than 12-years of education, the amount with more than the local average income, the amount with dental insurance, Caesarean section rate, PB rate and SGA rate between the PG and H groups, or the PLBW and HD groups. Furthermore, Pearson's correlation coefficient test was used to analyze the correlation between periodontal parameters and bacterial load.

To determine potential bacterial and periodontal risk factors for pregnant women, we evaluated correlations between the presence of periodontal disease or PLBW with PPD ≥ 5 mm (%), BOP, the duration of tooth brushing, the amount of *P. gingivalis*, *A. actinomycetemcomitans*, *T. forsythia*, *T. denticola*, *P. intermedia*, *Fretibacterium* sp. HOT 360, *R. dentocariosa,* *E. saphenum* in saliva and IgG against *A. actinomycetemcomitans* using ordinal regression analysis, respectively. Odds ratios and 95% confidence intervals were calculated. A *P* value less than 0.05 was considered statistically significant.

## Supplementary information

Supplementary Information 1.
