# Supplementary material for: Unculturable and culturable periodontal-related bacteria are associated with periodontal inflammation during pregnancy and with preterm low birth weight delivery
Source: Sci Rep. 2020 Sep 25;10:15807. doi: 10.1038/s41598-020-72807-9 (PMC7519089; doi:10.1038/s41598-020-72807-9)
Supplement: Supplementary file 1 — Supplementary Information 1. [file 41598_2020_72807_MOESM1_ESM.docx]

**Unculturable and culturable periodontal-related bacteria are associated with periodontal inflammation during pregnancy and with preterm low birth weight delivery**

Changchang Ye^1^, Zhongyi Xia^1^, Jing Tang^1^, Thatawee Khemwong^2^, Yvonne Kapila^3^, Ryutaro Kuraji^4^, Ping Huang^1^, Yafei Wu^1^, Hiroaki Kobayashi^2*^

^1^ State Key Laboratory of Oral Diseases & National Clinical Research Center for Oral Diseases & Dept. of Periodontology West China Hospital of Stomatology, Sichuan University, Chengdu, China

^2^Department of Periodontology, Graduate School of Medical and Dental Sciences, Tokyo Medical and Dental University (TMDU), Tokyo, Japan

^3^School of Dentistry, University of California San Francisco, San Francisco, California, USA

^4^Department of Life Science Dentistry and Department of Periodontology, The Nippon Dental University School of Life Dentistry at Tokyo, Tokyo, Japan

Corresponding author:

*Hiroaki Kobayashi, D.D.S., Ph.D.

Department of Periodontology, Graduate School of Medical and Dental Science, Tokyo Medical and Dental University (TMDU), Tokyo, Japan

1-5-45 Yushima, Bunkyo-ku, Tokyo, Japan 113-8549

Tel: +81-3-5803-5488/ Fax: +81-3-5803-0196

Email: [h-kobayashi.peri@tmd.ac.jp](mailto:h-kobayashi.peri@tmd.ac.jp)

| **Table S1. Serum IgG antibody against periodontal pathogens in Healthy and Periodontitis/Gingivitis groups** | | | | | |
| --- | --- | --- | --- | --- | --- |
|  | | H (n=20) | PG (n=70) | | *P*-value |
| IgG against *P. gingivalis* | 11.4 (9.0 12.6) | | 11.8 (11.0 12.4) | NS | |
| IgG against *A. actinomycetemcomitans* | 6.1 (5.3 9.0) | | 6.1 (5.2 10.1) | NS | |
| IgG against *T. denticola* | 9.8 (9.1 10.8) | | 10.0 (9.2 10.8) | NS | |
| IgG against *T. forsythia* | 11.6 (10.4 13.2) | | 12.1 (11.2 13.0) | NS | |
| IgG against *P. intermedia* | 9.6 (9.2 10.4) | | 9.8 (9.3 10.3) | NS | |
| IgG against *F. nucleatum* | 8.6 (7.1 10.4) | | 9.8 (7.5 10.2) | NS | |

Variables were tested using the Mann-Whitney U-test and are given as medians (quartile). ^*^Significantly different from the H

group (*P*<0.05)

| **Table S2 The amount of bacteria in saliva samples between PG and H groups.** | | | |
| --- | --- | --- | --- |
| Bacteria | H | PG | *P*-value |
| *P. gingivalis* | 3.3x10^3^(2.8x10 1.3x10^4^) | **1.5x10^4^ (1.0x10^3^ 4.7x10^4^) ^*^** | 0.048 |
| *A. actinomycetemcomitans* | 3.7x10 (2.6x10 5.5x10) | **5.2x10 (3.2x10 7.9x10) ^*^** | 0.048 |
| *T. denticola* | 4.8x10^3^ (6.8x10^2^ 1.4x10^4^) | **2.8x10^4^ (8.1x10^3^ 1.0x10^4^) ^*^** | 0.000 |
| *T. forsythia* | 3.5x103(6.8x10^2^ 1.1x10^4^) | **1.1x10^4^ (2.4x10^3^ 3.8x10^4^) ^*^** | 0.049 |
| *P. intermedia* | 2.6x10^3^ (6.9x10 2.8x10^4^) | **2.7x10^4^ (8.1x10^3^ 7.8x10^4^) ^*^** | 0.008 |
| *F. nucleatum* | 4.7x10^5^(9.6x10^4^ 1.6x10^6^) | 8.9x10^5^ (1.4x10^5^ 2.4x10^6^) | NS |
| *R. dentocariosa* | 7.9x10^3^ (1.9x10^3^ 4.0x10^4^) | **2.7x10^3^ (4.0x10^2^ 1.4x10^4^)^*^** | 0.032 |
| *Fretibacterium* sp. HOT 360 | 3.7x10^3^ (9.3x10^2^ 1.9x10^4^) | **2.6x10^4^ (8.3x10^3^ 5.4x10^4^) ^*^** | 0.003 |
| *TM7* sp. HOT356 | 8.4x10 (1.5x10 9.4x10^2^) | 3.3x10^2^ (4.5x10 2.6x10^3^) | NS |
| *E. saphenum* | 3.0x10^3^ (4.6 1.5x10^4^) | 4.2x10^3^ (2.7x10 2.4x10^4^) | NS |
| Variables were tested using the Mann-Whitney U-test and are given as medians (quartile). ^*^Significantly different from the H group (*P*<0.05) | | | |

| **Table S3.** **Correlation between periodontal parameters and bacterial load; adjusted for confounders** | | | | | | |
| --- | --- | --- | --- | --- | --- | --- |
| Bacteria | Mean PPD | | Mean CAL | | Percentage of BOP（+）sites | |
|  | *r* | *P-value* | *r* | *P-value* | *r* | *P-value* |
| *P. gingivalis* | 0.12 | NS | 0.13 | NS | 0.32 | 0.002 |
| *A. actinomycetemcomitans* | 0.04 | NS | 0.05 | NS | 0.25 | 0.017 |
| *T. denticola* | 0.26 | 0.014 | 0.24 | 0.02 | 0.411 | 0.000 |
| *T*. *forsythia* | 0.14 | NS | 0.15 | NS | 0.12 | NS |
| *P.* *intermedia* | 0.38 | 0.000 | 0.37 | 0.000 | 0.34 | 0.001 |
| *F. nucleatum* | 0.08 | NS | 0.07 | NS | -0.09 | NS |
| *R. dentocariosa* | -0.32 | 0.002 | -0.36 | 0.001 | -0.29 | 0.005 |
| *TM7* sp. HOT 356 | 0.13 | NS | 0.12 | NS | 0.15 | NS |
| *Fretibacterium* sp. HOT 360 | 0.25 | 0.02 | 0.25 | 0.02 | 0.32 | 0.002 |
| *E. saphenum* | 0.02 | NS | 0.003 | NS | -0.2 | NS |
| r: Pearson product-moment correlation coefficient. NS: not significant   \| **Table S4. Serum IgG antibody against periodontal pathogens in HD and PLBW groups** \| \| \| \| \| \| \| --- \| --- \| --- \| --- \| --- \| --- \| \|  \| \| HD (n=22) \| PLBW (n=22) \| \| *P*-value \| \| IgG against *P. gingivalis* \| 11.9 (10.5 12.5) \| \| 11.7 (10.4 11.9) \| NS \| \| \| IgG against *A. actinomycetemcomitans* \| 6.3 (5.4 10.1) \| \| 5.5 (4.8 8.1) \| NS \| \| \| IgG against *T. denticola* \| 9.9 (9.1 10.8) \| \| 10.0 (9.6 10.9) \| NS \| \| \| IgG against *T. forsythia* \| 12.0 (11.2 13.1) \| \| 11.8 (10.2 12.7) \| NS \| \| \| IgG against *P. intermedia* \| 9.8 (9.4 10.3) \| \| 9.5 (7.4 10.1) \| NS \| \| \| IgG against *F. nucleatum* \| 8.9 (7.5 10.4) \| \| 9.0 (7.4 10.2) \| NS \| \|   Variables were tested using the Mann-Whitney U-test and are given as medians (quartile). ^*^Significantly different from the HD  group (*P*<0.05)   \|  \| \| \| \| \| --- \| --- \| --- \| --- \| \| **Table S5. Comparison of the amount of bacteria in saliva samples for the PLBW and HD groups** \| \| \| \| \| Bacteria \| HD \| PLBW \| *P*-value \| \| *P. gingivalis* \| 1.2x10^4^ (3.7x10^2^ 5.2x10^4^) \| 6.5x10^3^ (3.0x10 2.2x10^4^) \| NS \| \| *A. actinomycetemcomitans* \| 4.8x10 (2.7x10 7.3x10) \| 4.0x10 (3.4x10 7.5x10) \| NS \| \| *T. denticola* \| 2.2x10^4^ (5.7x10^3^ 1.2x10^5^) \| 1.7x10^4^ (5.7x10^3^ 4.8x10^4^) \| NS \| \| *T. forsythia* \| 8.8x10^3^ (1.5x10^3^ 3.4x10^4^) \| 7.7x10^3^ (3.0x10^3^ 2.0x10^4^) \| NS \| \| *P. intermedia* \| 2.5x10^4^ (3.7x10^3^ 1.1x10^5^) \| 1.4x10^4^ (9.0x10^2^ 3.8x10^4^) \| NS \| \| *F. nucleatum* \| 7.9x10^5^ (1.0x10^5^ 2.2x10^6^) \| 1.1x10^6^ (1.3x10^5^ 2.5x10^6^) \| NS \| \| *R. dentocariosa* \| 3.4x10^3^ (5.4x10^2^ 1.8x10^4^) \| 2.2x10^3^ (6.1x10^2^ 1.2x10^4^) \| NS \| \| *TM7* sp. HOT356 \| 1.5x10^2^ (2.3x10 2.6x10^3^) \| 2.6x10^2^ (6.8x10 7.8x10^2^) \| NS \| \| *Fretibacterium* sp. HOT 360 \| 1.8x10^4^ (3.4x10^3^ 5.8x10^4^) \| 2.4x10^4^ (8.5x10^3^ 4.7x10^4^) \| NS \| \| *E. saphenum* \| 6.8x10^3^ (3.4x10 3.1x10^4^) \| **3.2x10 (6.4 5.6x10^3^) ^*^** \| 0.009 \| \| Variables were tested using the Mann-Whitney U-test and are given as medians (quartile). ^*^Significantly different from the HD  group (*P*<0.05) \| \| \| \| | | | | | | |

**Table S6. Correlation between birth results and bacterial load; adjusted for confounders**

|  | **Birth weeks (Gestational week at birth)** | | **Birth weight** | |  |
| --- | --- | --- | --- | --- | --- |
|  | *R* | *P-value* | *R* | *P-value* |  |
| *P. gingivalis* | 0.003 | NS | 0.1 | NS |  |
| *A. actinomycetemcomitans* | -0.04 | NS | -0.1 | NS |  |
| *T. denticola* | -0.03 | NS | 0.03 | NS |  |
| *T*. *forsythia* | -0.01 | NS | -0.01 | NS |  |
| *P.* *intermedia* | -0.08 | NS | 0.14 | NS |  |
| *F. nucleatum* | 0.03 | NS | -0.01 | NS |  |
| *R. dentocariosa* | | 0.03 | NS | 0.1 | NS |
| *TM7 sp. HOT 356* | | 0.02 | NS | -0.1 | NS |
| *Fretibacterium sp. HOT 360* | | -0.1 | NS | -0.1 | NS |
| *E. saphenum* | | 0.22* | 0.037 | 0.13 | NS |
| r: Pearson product-moment correlation coefficient. NS: not significant | | | | |  |
